# Developmental changes in activity-dependent plasticity of maturation-related neuronal phenotypes

**DOI:** 10.3389/fnsyn.2026.1926357

**Published:** 2026-10-15

**Authors:** Katsunori Kobayashi, Hiroki Imbe

**Affiliations:** 1 Department of System Neurophysiology, Wakayama Medical University, Wakayama, Japan; 2 Department of Pharmacology, Graduate School of Medicine, Nippon Medical School, Tokyo, Japan

**Keywords:** dentate gyrus, ECT, hippocampus, maturation, mossy fiber, neuropsychiatric disorders

## Abstract

Immaturity of neurons in the adult hippocampal dentate gyrus (DG) has been implicated in the pathophysiology of neuropsychiatric disorders. Pseudo-immaturity of DG neurons can be induced by neuronal hyperexcitation in adulthood. This “dematuration,” a reversal of maturation, could lead to aberrant neuronal maturation, if activated during development. However, it has not been directly tested whether dematuration can be induced in developing neurons. In the present study, we examined the effect of electroconvulsive treatment (ECT) on DG neurons of mice at several different developmental stages. Electrophysiological recordings were made using hippocampal slices. We analyzed frequency facilitation, a form of short-term synaptic plasticity, at the DG output mossy fiber synapses as an index of functional maturity of DG neurons. ECT had no significant effects on mossy fiber synaptic transmission at early postnatal ages, but it significantly reduced frequency facilitation at the postnatal age of ≥4 weeks, suggesting the induction of dematuration. Neuronal maturation marker analysis confirmed this result. The development of responsiveness to ECT was accompanied by maturation of N-methyl-D-aspartate receptor functions at the excitatory synaptic input onto DG neurons. Our results demonstrate that neuronal dematuration by ECT becomes inducible in postnatal 4 weeks in the DG of mice. Neuronal hyperexcitation such as seizures or alterations in N-methyl-D-aspartate receptor signaling around this developmental stage might affect proper maturation of DG neurons, thereby possibly increasing a risk of neuropsychiatric disorders.

## 1 Introduction

Dysregulated neuronal development and maturation have been implicated in the pathogenesis and pathophysiology of neuropsychiatric disorders (Lewis and Levitt, 2002; Sanches et al., 2008; Tavitian et al., 2019). Accumulating evidence suggests that the dentate gyrus (DG) of the hippocampus is critically involved in the pathophysiology of neuropsychiatric disorders including schizophrenia, epilepsy and depression (Kobayashi, 2009; Scharfman, 2019; Tavitian et al., 2019; Sun et al., 2023). In several lines of mouse models of neuropsychiatric disorders, immaturity of the granule cells (GCs) of the DG has been demonstrated (Yamasaki et al., 2008; Shin et al., 2013; Takao et al., 2013; Hagihara et al., 2022). The dentate GCs exhibit substantial developmental changes in their physiological and functional properties such as somatic excitability and synaptic plasticity (Ambrogini et al., 2004; Marchal and Mulle, 2004; Schmidt-Hieber et al., 2004; Kobayashi et al., 2010). Failure in maturation of the GCs can therefore lead to significant dysfunction in the adult hippocampal neuronal circuits. Molecular mechanisms underlying the dysregulation of neuronal maturation processes remain to be elucidated. In mice lacking Schnurri-2, a model of schizophrenia and intellectual disability, developmental changes in the expression of calbindin, a marker for mature GCs, were examined (Takao et al., 2013). The calbindin expression increases postnatally and reaches the mature level in 4–5 weeks in normally developing mice (Kobayashi et al., 2010). In Schnurri-2 mutant mice, the calbindin expression level was found to be similar to that of wild-type mice at the postnatal age of 2 weeks. The calbindin expression level further increased at 4 weeks in wild-type mice, but it decreased in the mutant mice (Takao et al., 2013). This finding suggests that the developmental period between postnatal 2 and 4 weeks, the middle to late maturation stage of the dentate GCs, may be critical for determining subsequent developmental trajectory of these neurons in mice.

Previous studies have revealed that the mature state of the dentate GCs can be changed into immature-like one in adult mice. This plasticity of the maturation status, named “dematuration,” was demonstrated in mice chronically treated with antidepressant drugs (Kobayashi et al., 2010, 2013, 2022; Imoto et al., 2015) and after repeated electroconvulsive treatment (ECT) (Imoto et al., 2017). Dematuration of the dentate GCs has been proposed as the neuronal substrate shared by chemical and physical antidepressant treatments. However, excessive dematuration in antidepressant-treated mice is associated with behavioral abnormality resembling manic switch caused by antidepressants (Kobayashi et al., 2011). Dematuration has also been demonstrated in a mouse model of temporal lobe epilepsy (Shin et al., 2013). Pseudo-immaturity of adult neurons caused by dematuration may therefore contribute to the pathophysiological basis of neuropsychiatric disorders in some conditions. Given the induction of dematuration by ECT and epileptic seizures, neuronal excitation is likely to play a key role in the plasticity of the neuronal maturation status in adults. Indeed, optogenetically-induced GC excitation has recently been shown to have the ability to induce dematuration (Murano et al., 2026). In SNAP-25 mutant mice, both spontaneous seizures and immaturity of the dentate GCs were observed (Kataoka et al., 2011; Ohira et al., 2013). Spontaneous seizures occur after postnatal day 21–24 in these mice, and antiepileptic drug treatment starting at postnatal day 16 rescued the immature phenotype of the GCs, which raises the possibility that neuronal excitation can affect the maturation status of the GCs in developing mice as well as in adults. The induction of dematuration during development would prevent proper maturation of the GCs and may contribute to the pathogenic process of neuropsychiatric disorders. However, no prior study has investigated activity-dependent reversal of neuronal maturation in young neurons. Epileptic seizures during the first few postnatal weeks do not usually cause lasting changes in the hippocampal circuit such as neuronal damage and synaptic reorganization (Galanopoulou and Moshé, 2009), indicating that young neurons are less sensitive to hyperexcitation in some respects. To understand pathogenic mechanisms of neuropsychiatric disorders associated with dysregulated neuronal maturation, it would be important to clarify the effects of hyperexcitation on young neurons at different developmental stages.

Here, we examine the effects of neuronal hyperexcitation on the maturation status of the dentate GCs using mice at postnatal week 2–9. We utilize ECT as a model of neuronal hyperexcitation and examine its effects at the identical stimulus intensity in mice with different developmental stages. Our results demonstrate that dematuration of the dentate GCs by ECT is established in postnatal 4 weeks.

## 2 Materials and methods

### 2.1 Animals and housing

Pregnant or neonatal C57BL/6J mice were purchased from Japan SLC. Mice were weaned between 3 and 4 weeks of age and housed in groups of 2–4. Only male mice were used in the present study. Adult mice (>8 weeks old) were purchased at the age of 7 weeks and housed in group. Mice were maintained in 14:10 or 12:12 light/dark cycle (lights on at 6:00 a.m. or 8:00 a.m. through 8:00 p.m.) at 23 °C ± 1 °C with food and water available ad libitum. A total of 83 mice were used in the present study. Animal use and procedures were in accordance with the National Institute of Health guidelines and approved by the Animal Care and Use Committees of Wakayama Medical University and Nippon Medical School.

### 2.2 Electroconvulsive treatment

We used ECT to induce neuronal hyperexcitation, but not to model electroconvulsive therapy for depression. Bilateral ECT (current, 15 mA; shock duration, 1 s; frequency, 100 pulse/s; pulse width, 0.5 ms) was administered via moistened, spring-loaded ear-clip electrodes with a pulse generator (ECT Unit; Ugo Basile, Italy) to mice at different developmental stages (Figure 1). Each mouse received three rounds of ECT in 4 or 5 days. Mice as young as ≤2 weeks were used in the present study, so the current intensity of ECT was smaller than that used previously to induce dematuration in adults (Imoto et al., 2017). The ECT parameter was the same across ages, although there was an approximately 2-fold weight difference between younger and older mice. Seizure duration may vary across ages. ECT was suggested in a pilot study to be less effective in inducing GC dematuration in younger mice (see Figure 2). To exclude the possibility that the failure in the induction of dematuration in younger mice is due to insufficient stimulus intensity, we used the same stimulus intensity for all mice. Mice were anesthetized with isoflurane (1.5%–2%) to avoid sudden unexpected death associated with seizures. None of mice died in the present treatment condition. The sham-treated control mice were anesthetized with isoflurane for the same duration and handled in an identical manner to the ECT-treated mice, but without the administration of electrical shock. In most cases, littermates were assigned to ECT or sham treatment at each developmental stage. Experimenters were not blind to the treatment. In the experiments shown in Supplementary Figure 1, ECT was delivered at the current intensity of 25 mA and repeated twice. Hippocampal slices for electrophysiological experiments and PCR were prepared about 24 h after the last ECT or sham treatment.

**FIGURE 1:**
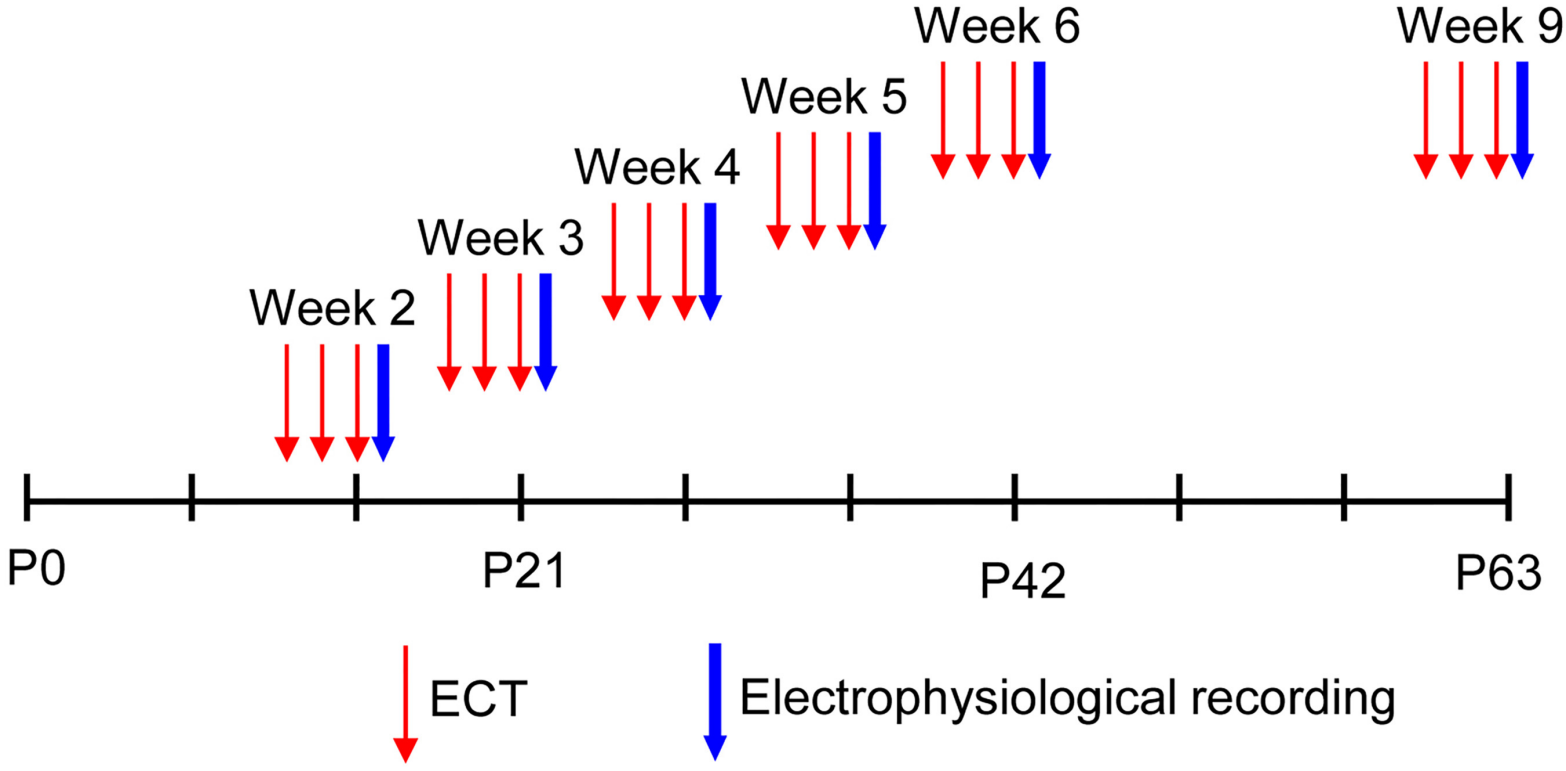
Schematic diagram showing timeline of electroconvulsive treatment (ECT) and electrophysiological recording.

**FIGURE 2:**
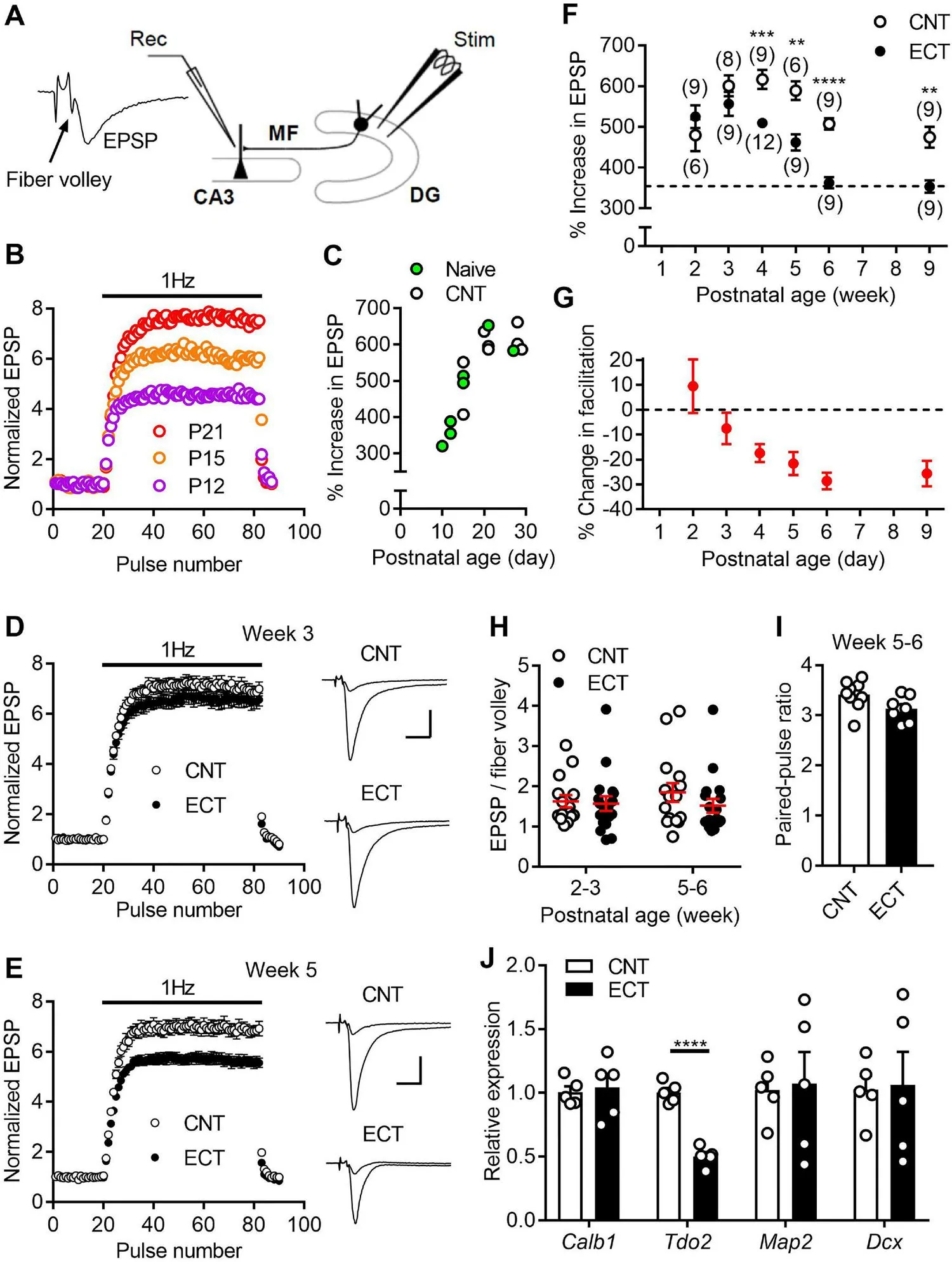
Effects of electroconvulsive treatment (ECT) on maturation status of developing granule cells. **(A)** An example of field excitatory postsynaptic potential (EPSP) at the mossy fiber (MF)-CA3 synapse and a diagram showing the electrode arrangement for recording EPSPs. **(B)** Frequency facilitation at the MF synapse induced by 1 Hz stimulation at different postnatal ages. **(C)** Developmental increase in the magnitude of frequency facilitation in naive mice and sham-treated control mice (CNT). **(D,E)** Effects of ECT on frequency facilitation at postnatal week 3 **(D)** and 5 **(E)** . Sample recordings are averages of 10 consecutive EPSPs during baseline and 1 Hz stimulation. Scale bars: 10 ms and 0.5 mV. **(F)** Developmental changes in frequency facilitation and the effects of ECT on frequency facilitation. The horizontal dashed line represents the average magnitude of facilitation in 10–12 days old mice shown in panel **(C)** . The number of data is shown in parentheses. Week 4 (t_19_ = 4.494, ***p = 0.0002), week 5 (t_13_ = 4.098, **p = 0.0013), week 6 (t_16_ = 7.31, ****p < 0.0001), week 9 (Mann Whitney test, **p = 0.0019). Effect sizes with 95% confidence intervals: week 2, 0.51 [−0.55, 1.55]; week 3, 0.55 [−0.43, 1.51]; week 4, 1.98 [0.9, 3.03]; week 5, 2.16 [0.81, 3.45]; week 6, 3.45 [1.92, 4.93]; week 9, 1.91 [0.75 to 3.02]. **(G)** Developmental increase in the effect of ECT on frequency facilitation. ECT-induced changes in the magnitude of facilitation relative to control mice at each age were calculated using data shown in panel **(F)** . **(H)** Effects of ECT on EPSP to fiber volley ratios in early and late postnatal developmental periods. **(I)** Effects of ECT on paired-pulse facilitation ratios (50 ms interval) in late postnatal developmental period. **(J)** Effects of ECT on expression levels of GC maturation stage markers at postnatal week 6. *Tdo2* (t_18_ = 10.17, ****p < 0.0001).

### 2.3 Electrophysiological analysis

Mice were decapitated under deep isoflurane anesthesia, and both hippocampi were isolated. Transverse slices (300–380 μm) were cut from the middle part of the hippocampus using a tissue slicer (7000smz, Campden Instruments Ltd., Leics., UK) in ice-cold saline (see below). Slices were then incubated for 30 min at 30 °C and maintained in a humidified interface holding chamber at room temperature before use. Electrophysiological recordings were made in a submersion-type chamber maintained at 27.0 °C–27.5 °C and superfused at 2 ml/min with recording saline composed of (in mM): NaCl, 125; KCl, 2.5; NaH_2_PO_4_, 1.0; NaHCO_3_, 26.2; glucose, 11; CaCl_2_, 2.5; MgCl_2_, 1.3 (equilibrated with 95% O_2_/5% CO_2_). Field excitatory postsynaptic potentials (EPSPs) arising from the mossy fiber (MF) synapses were evoked by stimulating the dentate GC layer with bipolar tungsten electrodes and recorded from the stratum lucidum of the CA3 region using a glass pipette filled with 2 M NaCl. The amplitude of field EPSPs was measured as described (Kobayashi and Suzuki, 2007; Kobayashi et al., 2010). A criterion used to identify the MF input was more than 85% block of EPSPs by an agonist of group II metabotropic glutamate receptors, (2S,2′R,3′R)-2-(2′,3′-dicarboxycyclopropyl)glycine (DCG-IV, 1 μM). Single electrical stimulation was delivered at a frequency of 0.05 Hz unless otherwise stated. For recording field potentials at the synapses between the medial perforant path (MPP) and GCs, both stimulating and recording electrodes were placed in the middle third of the molecular layer of the DG. To record EPSPs mediated by N-methyl-D-aspartate (NMDA) receptors, the extracellular concentration of MgCl_2_ was reduced to 0.2 mM, and 6-cyano-7-nitroquinoxaline-2,3-dione (CNQX, 10 μM) and picrotoxin (0.1 mM) were added to the bath. The initial slope and amplitude of EPSPs were measured for recordings in the normal and low Mg^2+^ saline, respectively. All recordings were made using a Multiclamp 700B amplifier (Molecular Devices, Sunnyvale, CA, USA), filtered at 2 kHz and stored in a personal computer via an interface (digitized at 10 kHz). DCG-IV, D-2-amino-5-phosphonovalerate (D-APV) and CNQX were purchased from Tocris Bioscience (Bristol, UK). Picrotoxin was purchased from Fujifilm Wako Pure Chemical Industries (Osaka, Japan). 3-(2-carboxypiperazin-4yl) propyl-1-phosphonic acid (CPP) was purchased from Sigma-Aldrich (St. Louis, MO, USA).

### 2.4 Real time PCR

Total RNA was isolated from the DG dissected out of the hippocampal slice using RNeasy Lipid Tissue Mini Kit (QIAGEN, Venlo, Netherlands). The total RNA was converted into cDNA with PrimeScriptTMRT reagent Kit with gDNA Eraser (RR047A, Takara, Kusatsu, Japan). Quantitative RT-PCR was performed using TB Green Premix Ex TaqTM II (RR820A, Takara) and a Real Time System Single (Thermal Cycler Dice, TP850, Takara). Gene expression levels were calculated by the delta-delta Ct method and normalized to 18S rRNA. The sequences of the primers used were as follows:

Calb1 forward, 5′-TCTGGCTTCATTTGGACGCTG-3′;

Calb1 reverse, 5′-ACAAAGGATTTCATTTCCGGTGA-3′;

Tdo2 forward, 5′-ATGAGTGGGTGCCCGTTTG-3′;

Tdo2 reverse, 5′-GGCTCTGTTTACACCAGTTTGAG-3′;

Dcx forward, 5′-GATGTCCCTGATCCCTGCATTAC -3′;

Dcx reverse, 5′TGCTGACTGGAGCCTAGCTGA -3′;

Map2 forward, 5′-CCACGTACCTGGAGGTGGTAATG -3′;

Map2 reverse, 5′-GTGATCTACCCGGGCCTTTG -3′;

18S rRNA forward, 5′-GAGGCCCTGTAATTGGAATGAG-3′;

18S rRNA reverse, 5′-GCAGCAACTTTAATATACGCTA TTGG-3′.

### 2.5 Statistics

All data are presented as means ± SEM. The number of data represents the number of slices and mice in electrophysiology and RT-PCR, respectively. The required sample size in the ECT experiments was estimated using G*Power3.1.9.7 based on the effect size (Cohen’s d, 2.34) in our previous experiment using two rounds of ECT (Imoto et al., 2017). It was calculated that six samples for each group were required to obtain effects comparable to the previous results (α = 0.05, 1−β = 0.95). Effect sizes and confidence intervals were calculated using the effect size package of R. Other statistical analyses were performed using GraphPad Prism version 7.01. Experiments with two groups were compared using unpaired two-tailed Student’s *t*-test unless otherwise specified. Experiments with more than two groups were subjected to one-way ANOVA or two-way ANOVA, followed by the Holm-Sidak’s multiple comparisons test. Data that did not pass the Shapiro-Wilk normality test were analyzed using nonparametric tests. Statistical significance was set at *p* < 0.05.

## 3 Results

A characteristic functional feature of the mature dentate GCs is marked frequency facilitation, an activity-dependent presynaptic form of short-term plasticity, at their output MF synapses. To evaluate the effect of ECT on the maturation status of GCs during postnatal development, we first focused on the MF synaptic transmission (Figure 2A). Frequency facilitation at the MF synapses rapidly increases by about postnatal 4 weeks (Figures 2B, C; Marchal and Mulle, 2004; Kobayashi et al., 2010), which parallels an increase in the expression levels of mature GC markers (Kobayashi et al., 2010). Frequency facilitation then slightly decreases toward the mature level (Figure 2F; Mori-Kawakami et al., 2003; Kobayashi et al., 2010). In adult mice, chronic antidepressant treatments and ECT reduce the magnitude of frequency facilitation to the juvenile level (Kobayashi et al., 2010; Imoto et al., 2017). As in the previous study, ECT repeated three times reduced frequency facilitation in 9-week-old mice to the juvenile level (Figure 2F). This treatment paradigm was then applied to developing mice (Figure 1). ECT had no significant effects in 3-week-old mice (Figure 2D), but it significantly reduced frequency facilitation in 5-week-old mice (Figure 2E). The magnitude of facilitation in control mice was similar between these ages. A significant reduction of facilitation was observed at the age of 4 weeks, and the magnitude of the reduction reached that of adult ECT-treated mice at 6 weeks (Figure 2G).

In general, synaptic facilitation decreases with an increase in the baseline transmitter release probability (Zucker and Regehr, 2002). The basal synaptic efficacy was evaluated by analyzing ratios of EPSPs to presynaptic fiber volleys (Figure 2A). There was no clear change in the EPSP to fiber volley ratios during development, and ECT had no significant effect on the ratios in either early or late developmental stage (Figure 2H). In addition, paired-pulse facilitation, another form of short-term plasticity, was not significantly changed by ECT (Figure 2I). The ECT-induced reduction in frequency facilitation in the late developmental period cannot therefore be simply explained by an increase in the basal synaptic efficacy. Rather, it likely represents dematuration of GCs, as previously demonstrated in adult mice (Imoto et al., 2017). To confirm this idea, we examined the expression levels of the genes encoding calmodulin (*Calb1*) and tryptophan 2,3-dioxygenase (*Tdo2*), molecular markers of mature GCs (Kobayashi et al., 2010; Ohira et al., 2010). Effects of ECT on the expression of these genes were well characterized in adult mice (Imoto et al., 2017). In 6-week-old mice, ECT strongly reduced the expression level of *Tdo2*, while it did not change the *Calb1* expression (Figure 2J). The higher sensitivity of the *Tdo2* expression level to ECT is consistent with the previous study (Imoto et al., 2017). We suggest the lack of change in the *Calb1* expression is probably due to the lower stimulus intensity of ECT used in the present study (see section “2 Materials and methods”). In addition, the repetition of ECT may be insufficient for suppressing the *Calb1* expression (see section “4 Discussion”). We also examined the expression of doublecortin (*Dcx*), a marker for neuronal progenitors and early immature neurons, and microtubule-associated protein 2 (*Map2*), a neuronal marker abundant in the adult brain, and found no significant effects of ECT (Figure 2J), which excludes the possibility of non-specific downregulation of gene expression by ECT. Taken together, these results suggest that the inducibility of dematuration by ECT emerges by the age of 4 weeks during the postnatal development in mice.

The induction of dematuration by ECT in adults requires activation of NMDA-type glutamate receptors (Imoto et al., 2017). Antagonists of NMDA receptors can block ECT-induced downregulation of mature GC markers (Imoto et al., 2017) and attenuation of frequency facilitation at the MF synapses (Supplementary Figure 1). To get some mechanistic insight into the developmental change in the effectiveness of ECT, we examined the properties of the excitatory synaptic input onto the GCs. The MPP input was stimulated, and field EPSPs were recorded in the DG (Figure 3A). First, the input-output relationship at the MPP synapses was examined by varying the stimulus intensity. In young mice, electrical stimulation evoked slightly larger fiber volleys and smaller EPSPs as compared with adult mice (Figure 3B). The input-output relationship was clearly different between young and adult mice (Figure 3B). The basal excitatory synaptic transmission is mainly mediated by α-amino-3-hydroxy-5-methyl-4-isoxazolepropionic acid (AMPA) receptors. To accurately measure the fiber volley amplitude, AMPA receptors were blocked by CNQX (Figure 3C). The EPSP to fiber volley ratios of AMPA receptor-mediated responses significantly increased during postnatal development (Figure 3D). To evaluate functions of NMDA receptors, extracellular Mg^2+^ concentrations were lowered after adding CNQX. In the low Mg^2+^ solution, MPP stimulation evoked small EPSPs that were completely blocked by the NMDA receptor antagonist D-APV (25 μM) (Figure 3C). The ratios of NMDA receptor-mediated EPSPs to fiber volleys also increased during postnatal development (Figure 3E). These results indicate that the developmental increase in the effectiveness of ECT is associated with an increase in the efficacy of the glutamatergic synaptic input to the GCs.

**FIGURE 3:**
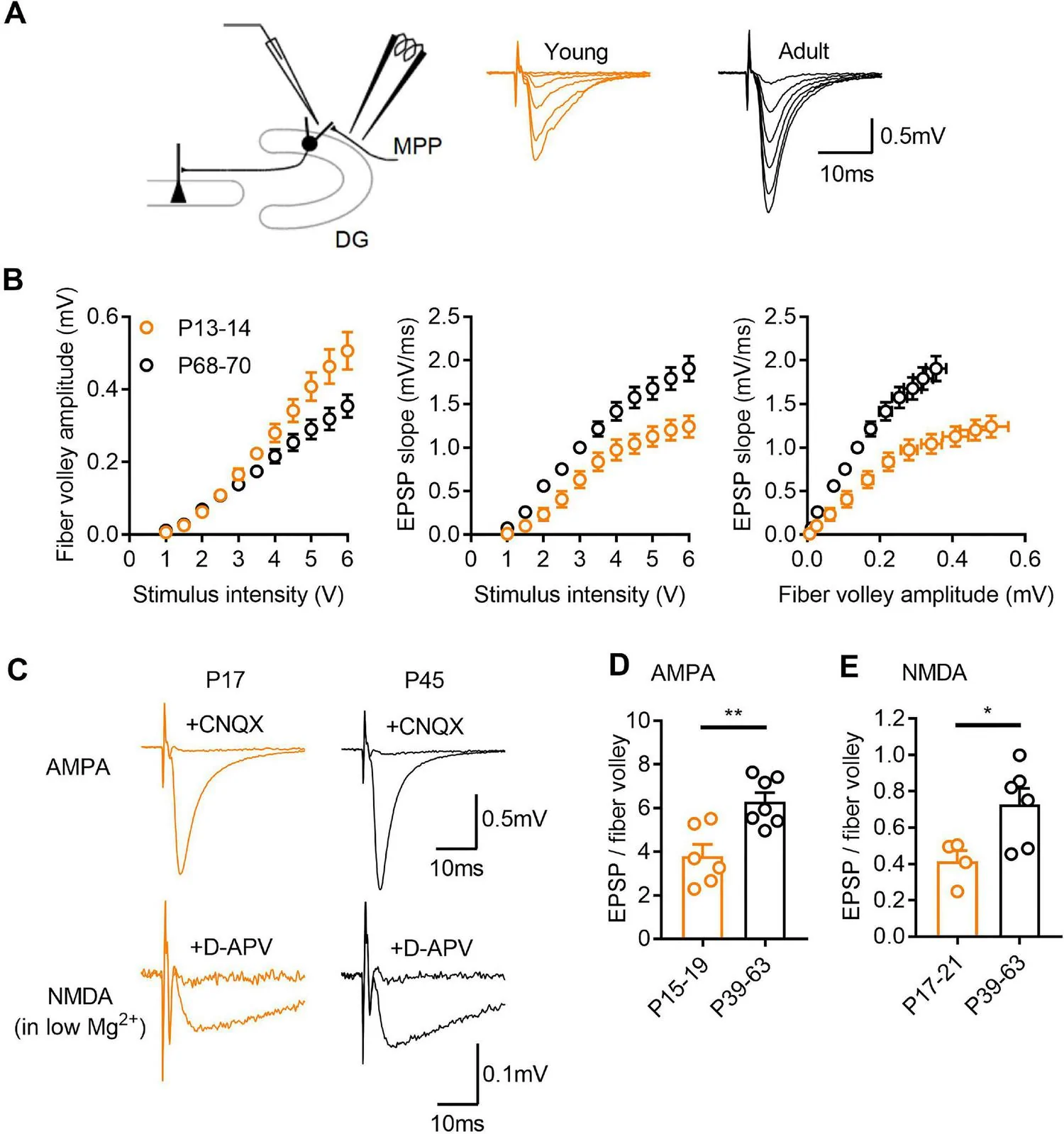
Developmental changes in glutamatergic input to granule cells. **(A)** A diagram showing the electrode arrangement for recording excitatory postsynaptic potentials (EPSPs) at medial perforant path (MPP)-dentate gyrus (DG) synapse and sample recordings of EPSPs evoked at different stimulus intensities. **(B)** Fiber volley amplitude (left) and EPSP slope (center) at different stimulus intensities, and input-output relationships (right) in young (*n* = 6) and adult (*n* = 5) mice. **(C)** α-amino-3-hydroxy-5-methyl-4-isoxazolepropionic acid (AMPA) receptor and N-methyl-D-aspartate (NMDA) receptor-mediated EPSPs in early and late postnatal periods. **(D)** Developmental increase in AMPA EPSP to fiber volley ratios (t_11_ = 3.735, **p = 0.0033). **(E)** Developmental increase in NMDA EPSP to fiber volley ratios (t_8_ = 2.619, *p = 0.0307).

Electroconvulsive treatment is supposed to induce high-frequency activation of the glutamatergic input to the GCs. We finally examined a possible difference in the responsiveness of the GCs to high-frequency synaptic activation between young and adult mice. High-frequency stimulation (HFS) of glutamatergic input induces long-term potentiation of synaptic transmission at many synapses. However, in the presence of intact synaptic inhibition, HFS of the MPP input barely induces lasting potentiation in slice preparations (Wigström and Gustafsson, 1983; Arima-Yoshida et al., 2011; Hayashi et al., 2014). In the present recording condition, HFS induced transient post-tetanic potentiation (PTP), but no lasting change in MPP EPSPs in adults (Figure 4A). In young mice, the magnitude of PTP was larger than that in adult mice (Figures 4A, B). Furthermore, HFS induced small long-term depression (LTD) in young mice, especially at the age of ≤2 weeks (Figures 4A, C). This LTD, but not PTP, was blocked by the NMDA receptor antagonist D-APV (Figures 4B, C). These results suggest that NMDA receptor-dependent signaling activated by HFS of glutamatergic input to the dentate GCs changes during initial weeks of postnatal development.

**FIGURE 4:**
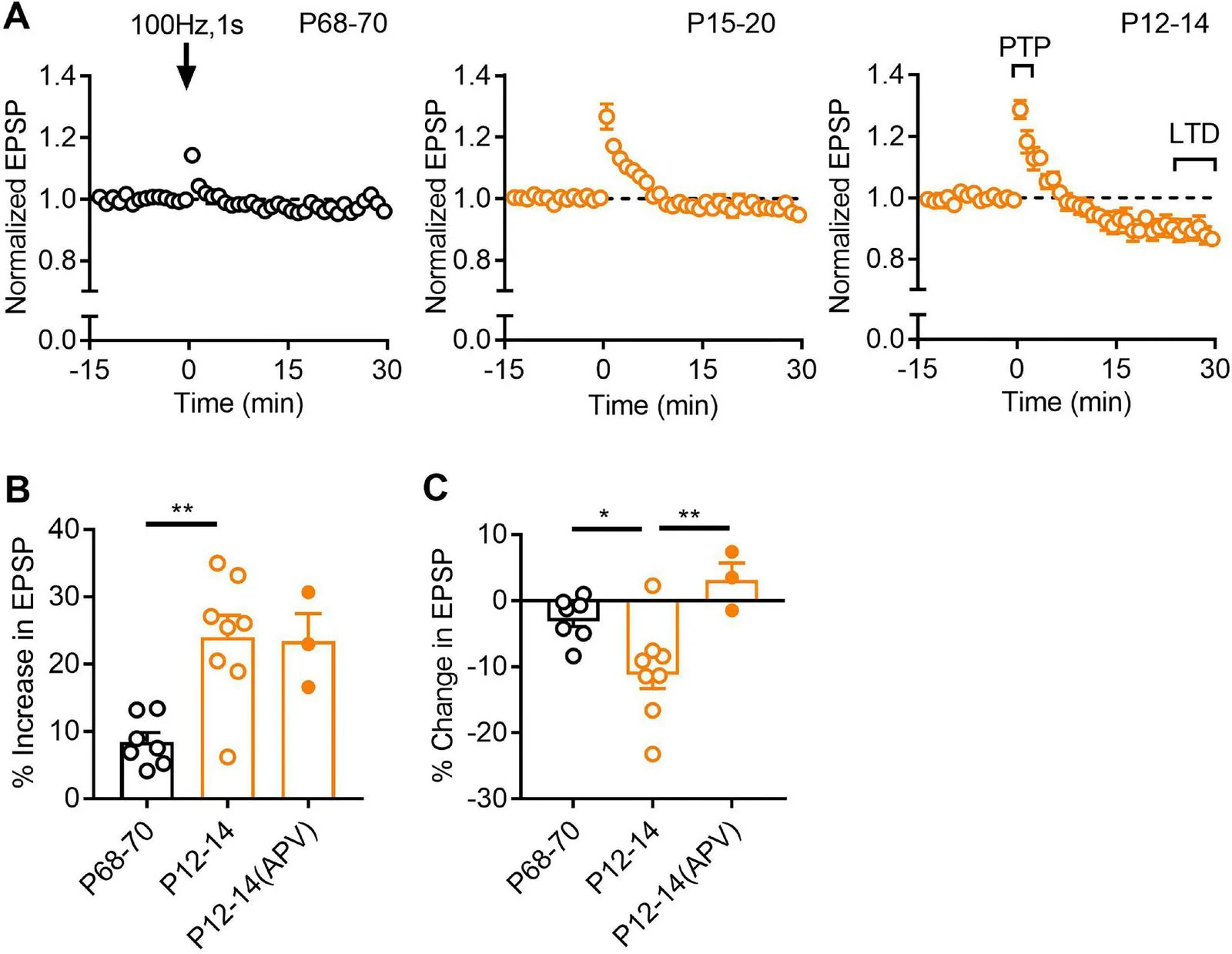
Developmental changes in synaptic plasticity induced by high-frequency stimulation of glutamatergic input to granule cells. **(A)** Effects of high-frequency stimulation (HFS) (100 Hz, 1 s) on synaptic transmission at different postnatal ages. HFS was applied at time 0. P68-70 (*n* = 7), P15-20 (*n* = 7), P12-14 (*n* = 8). **(B)** The magnitude of post-tetanic potentiation (PTP) measured at the time window indicated in panel **(A)** (one-way ANOVA, F_2,15_ = 10.1, *p* = 0.0017; followed by Holm-Sidak’s test, **p = 0.0014). **(C)** The magnitude of LTD measured at the time window indicated in panel **(A)** (one-way ANOVA, F_2,15_ = 7.662, *p* = 0.0051; followed by Holm-Sidak’s test, *p = 0.0156, **p = 0.0053).

## 4 Discussion

In the present study, we have demonstrated that the activity-dependent plasticity of the maturation-related phenotypes of the dentate GCs is established in 4–6 weeks of postnatal development in mice. Three rounds of ECT starting after the third postnatal week significantly reduced frequency facilitation at the MF synapse, which suggests dematuration of the GCs as demonstrated previously in adult mice (Imoto et al., 2017). In younger mice, ECT had no significant effect. The analysis of expression levels of neuronal maturation marker molecules also suggested a change in the maturation status of the GCs after ECT. The expression of the mature GC marker *Calb1* was not changed in the present experimental condition. In adult mice, the *Calb1* expression is immediately reduced by single ECT and recovers to the baseline level in 24 h. Repetition of ECT more than 10 times induces persistent downregulation of the *Calb1* expression and stable dematuration (Imoto et al., 2017). The present stimulus condition is insufficient for inducing stable dematuration, and the *Calb1* expression most likely recovered to the baseline at the time of sampling. Neuronal activity has been shown to promote maturation of the GCs at early maturational stages (Ge et al., 2006; Overstreet-Wadiche et al., 2006). Conversely, dematuration of the GCs can be induced at late maturational stages. Neuronal activity thus appears to have bidirectional effects on the maturation status of the developing GCs in a manner that is dependent on the maturational stage of the cells. Aberrant maturation of the dentate GCs has been implicated in a pathophysiological basis of neuropsychiatric disorders. Further investigation of activity-dependent bidirectional regulation of GC maturation during development may provide a novel insight into the pathogenic mechanisms of these disorders.

Immaturity of the dentate GCs has been demonstrated in several lines of mouse models of neuropsychiatric disorders (Yamasaki et al., 2008; Shin et al., 2013; Takao et al., 2013; Hagihara et al., 2022) and proposed as the pathophysiological basis of schizophrenia (Tavitian et al., 2019). In Schnurri-2 knockout mice, which exhibit substantial behavioral abnormalities related to schizophrenia, neuronal maturation processes appear to be reversed between postnatal week 2 and 4 (Takao et al., 2013). This time window matches the age at which ECT becomes effective in inducing dematuration as demonstrated here. Endogenous neuronal excitation does not seem to cause spontaneous dematuration of the GCs in normally developing mice. However, augmented neuronal excitation in some pathological conditions may lead to dematuration during development, resulting in aberrant maturation in adulthood. Neuronal inflammation was demonstrated in the brain of Schnurri-2 knockout mice (Takao et al., 2013) and has been implicated in the pathophysiology of schizophrenia (Chaves et al., 2024). Inflammation can increase neuronal excitability (Iori et al., 2016), thereby possibly impeding proper maturation of the GCs via facilitated induction of dematuration during development.

Dematuration of the GCs by ECT requires NMDA receptor activation (Imoto et al., 2017). The postnatal development of the ECT effect was associated with an increase in NMDA receptor-mediated synaptic responses and a decrease in NMDA receptor-dependent LTD. We therefore speculate that maturation of NMDA receptor-dependent signaling could be linked to inducibility of dematuration by ECT. In young mice, the activation level of NMDA receptors might be simply insufficient for the induction of dematuration. An alternative hypothesis is that intracellular signaling pathways that link NMDA receptor activation with the induction of dematuration are not fully developed. High-frequency stimulation of the MPP input induced NMDA receptor-dependent LTD in young mice, but not in adults. In the CA1 region of the hippocampus, NMDA receptor-dependent LTD has been reported to be regulated by many intracellular signaling molecules including calcineurin (Mulkey et al., 1994), α-Ca^2+^/calmodulin-dependent protein kinase II (Coultrap et al., 2014), cyclin-dependent protein kinase 5 (Ohshima et al., 2005), and glycogen synthase kinase-3 (Peineau et al., 2009). All of these signaling molecules have been implicated in regulation of GC maturation (Jessberger et al., 2008; Yamasaki et al., 2008; Pallas-Bazarra et al., 2017; Hagihara et al., 2022). In young mice, high-frequency activation of NMDA receptors by ECT may robustly activate some of these signaling pathways in the GCs, which may affect the process of dematuration. Hypofunction of NMDA receptors has long been implicated in the pathophysiology of schizophrenia (Nakazawa and Sapkota, 2020). This hypothesis originated from the observation that acute administration of an antagonist of NMDA receptors induces behaviors resembling symptoms of schizophrenia (Krystal et al., 1994), suggesting a direct link between NMDA receptor hypofunction and the symptom onset. Our present findings imply that altered NMDA receptor signaling during development can contribute to the pathogenic process of neuropsychiatric disorders. In this context, hyperfunction of NMDA receptors may promote aberrant induction of dematuration during development, thereby possibly being pathogenic. In support of this idea, mutant mice with DG-specific NMDA receptor hyperfunction exhibit a working memory deficit (Hayashi et al., 2014), a cognitive symptom observed in neuropsychiatric disorders including schizophrenia. Detailed analysis of the role of NMDA receptors in dematuration during development may reveal possible multiple roles of these receptors in the pathogenesis and pathophysiology of schizophrenia and related disorders.

Immaturity or dematuration of the GCs has been demonstrated in pilocarpine-induced epilepsy model mice (Shin et al., 2013) and in SNAP-25 mutant mice that exhibit epileptic seizures (Ohira et al., 2013). In the DG of patients with epilepsy, there has been an observation of reduction in the expression of calbindin, one of mature GC markers (Maglóczky et al., 1997; Martinian et al., 2012), which implies the altered maturation status of the GCs in human epilepsy as well. In SNAP-25 mutant mice, spontaneous seizures develop after postnatal day 21–24 (Kataoka et al., 2011). In general, status epilepticus in the young brain is less likely to cause subsequent spontaneous seizures and hippocampal neuronal damage as compared with that in the adult brain (Holmes et al., 1998; Galanopoulou and Moshé, 2009). This suggests postnatal development of neuronal plasticity that links acute seizures to lasting changes in the hippocampal neurons. Our results suggests that dematuration of the GCs plays a role in this plasticity. ECT failed to induce GC dematuration before postnatal day 21. Immature or “demature” GCs generally exhibit increased somatic excitability (Ambrogini et al., 2004; Schmidt-Hieber et al., 2004; Yamasaki et al., 2008; Kobayashi et al., 2010; Ohira et al., 2013; Shin et al., 2013; Takao et al., 2013; Imoto et al., 2017), and repeated ECT causes persistent dematuration (Imoto et al., 2017). The induction of GC dematuration would therefore theoretically promote subsequent epileptic discharge and the transition to chronic epilepsy. Status epilepticus during postnatal development can increase an incidence of epilepsy later in life (Kubová et al., 2004). In adult mice, activation of NMDA receptors is required for the maintenance of dematuration as well as its induction. The maintenance of the demature state of the GCs can be perturbed by antagonizing NMDA receptors after delivering ECT (Imoto et al., 2017). Understanding the NMDA receptor signaling involved in dematuration of developing GCs may provide a novel therapeutic target to prevent epilepsy that is promoted by seizures during early life.

A comparison of key events in human and rodent brain development suggests that the developmental period investigated in the present study roughly corresponds to infancy through adolescence in humans (Zeiss, 2021). To be more specific, while the calbindin expression in the DG reaches mature levels around postnatal week 5 in mice (Kobayashi et al., 2010; Radic et al., 2017), an adult-like calbindin expression pattern has been observed at 11 years of age in humans (Abrahám et al., 2009). The robust effects of ECT on GCs was first observed at postnatal week 4 in mice. Assuming similar activity-dependent plasticity in the human DG, our results suggest that neuronal hyperexcitation may cause dematuration of human GCs around the age of 10 years or older. The onset of idiopathic generalized epilepsies (IGEs) peaks around the age of 15 years (Asadi-Pooya et al., 2012). The developmental increase in the induction of GC dematuration may be relevant to the onset of IGEs. As discussed above, the induction of GC dematuration is predicted to promote the chronic progression of epilepsy. Therapeutic treatments targeting GC dematuration would help to prevent chronic epilepsy. Valproate has long been used to treat IGEs, although the mechanisms of its therapeutic effects are not fully understood. Valproate is also used for bipolar disorder and migraines, and several intracellular signaling pathways have been suggested as potential targets (Rosenberg, 2007). Modulation of intracellular signaling by valproate may contribute to its antiepileptic effect, especially during prolonged treatment. Valproate completely blocks the immature GC phenotypes of SNAP-25 mutant mice (Ohira et al., 2013). Although this effect is likely ascribed to suppression of spontaneous seizures in these mice, valproate may also have more direct effects on signaling pathways for regulation of GC maturation. Testing this possibility for valproate and other currently used antiepileptic drugs could be a promising way to find a novel target for treatment and prevention of IGEs.

There are a number of limitations that warrant mention in relation to this study. First, it was performed only using male mice, so any sex-specific mechanisms involved in excitation-induced plasticity of GC phenotypes and its developmental changes remain unknown. Also, the current intensity of ECT was the same across ages and was not normalized by body sizes (see section “2 Materials and methods”). Possible differences in seizure induction across ages may have affected the results.

In summary, our results demonstrate that dematuration of the dentate GCs by ECT is established around the postnatal age of 4 weeks in mice. Maturation of NMDA receptor signaling is a candidate process responsible for the developmental changes in the effects of ECT, although further investigation is needed to test this hypothesis. Neuronal hyperexcitation or alterations in N-methyl-D-aspartate receptor signaling at this late developmental stage may perturb proper maturation of DG neurons, thereby possibly increasing a risk of neuropsychiatric disorders such as schizophrenia and epilepsy. Signaling cascades involved in the induction of dematuration are potential therapeutic targets for preventing the pathogenesis of these disorders.

## Data Availability

The raw data supporting the conclusions of this article will be made available by the authors, without undue reservation.

## References

[B1] Abrahám H. Veszprémi B. Kravják A. Kovács K. Gömöri E. Seress L. (2009). Ontogeny of calbindin immunoreactivity in the human hippocampal formation with a special emphasis on granule cells of the dentate gyrus. *Int. J. Dev. Neurosci* 27, 115–127. 10.1016/j.ijdevneu.2008.12.004 19150647

[B2] Ambrogini P. Lattanzi D. Ciuffoli S. Agostini D. Bertini L. Stocchi V. et al. (2004). Morpho-functional characterization of neuronal cells at different stages of maturation in granule cell layer of adult rat dentate gyrus. *Brain Res*. 1017, 21–31. 10.1016/j.brainres.2004.05.039 15261095

[B3] Arima-Yoshida F. Watabe A. M. Manabe T. (2011). The mechanisms of the strong inhibitory modulation of long-term potentiation in the rat dentate gyrus. *Eur. J. Neurosci*. 33, 1637–1646. 10.1111/j.1460-9568.2011.07657.x 21535245

[B4] Asadi-Pooya A. A. Emami M. Sperling M. R. (2012). Age of onset in idiopathic (genetic) generalized epilepsies: Clinical and EEG findings in various age groups. *Seizure* 21, 417–421. 10.1016/j.seizure.2012.04.004 22560254

[B5] Chaves C. Dursun S. M. Tusconi M. Hallak J. E. C. (2024). Neuroinflammation and schizophrenia - is there a link? *Front. Psychiatry*. 15:1356975. 10.3389/fpsyt.2024.1356975 38389990 PMC10881867

[B6] Coultrap S. J. Freund R. K. O’Leary H. Sanderson J. L. Roche K. W. Dell’Acqua M. L. et al. (2014). Autonomous CaMKII mediates both LTP and LTD using a mechanism for differential substrate site selection. *Cell Rep*. 6, 431–437. 10.1016/j.celrep.2014.01.005 24485660 PMC3930569

[B7] Galanopoulou A. S. Moshé S. L. (2009). The epileptic hypothesis: Developmentally related arguments based on animal models. *Epilepsia* 50, 37–42. 10.1111/j.1528-1167.2009.02217.x 19682049 PMC2736106

[B8] Ge S. Goh E. L. Sailor K. A. Kitabatake Y. Ming G. L. Song H. (2006). Regulates synaptic integration of newly generated neurons in the adult brain. *Nature* 439, 589–593. 10.1038/nature04404 16341203 PMC1420640

[B9] Hagihara H. Shoji H. Kuroiwa M. Graef I. A. Crabtree G. R. Nishi A. et al. (2022). Forebrain-specific conditional calcineurin deficiency induces dentate gyrus immaturity and hyper-dopaminergic signaling in mice. *Mol. Brain*. 15:94. 10.1186/s13041-022-00981-0 36414974 PMC9682671

[B10] Hayashi Y. Nabeshima Y. Kobayashi K. Miyakawa T. Tanda K. Takao K. et al. (2014). Enhanced stability of hippocampal place representation caused by reduced magnesium block of NMDA receptors in the dentate gyrus. *Mol. Brain*. 7:44. 10.1186/1756-6606-7-44 24893573 PMC4073519

[B11] Holmes G. L. Gairsa J. L. Chevassus-Au-Louis N. Ben-Ari Y. (1998). Consequences of neonatal seizures in the rat: Morphological and behavioral effects. *Ann. Neurol*. 44, 845–857. 10.1002/ana.410440602 9851428

[B12] Imoto Y. Kira T. Sukeno M. Nishitani N. Nagayasu K. Nakagawa T. et al. (2015). Role of the 5-HT4 receptor in chronic fluoxetine treatment-induced neurogenic activity and granule cell dematuration in the dentate gyrus. *Mol. Brain*. 8:29. 10.1186/s13041-015-0120-3 25976618 PMC4430984

[B13] Imoto Y. Segi-Nishida E. Suzuki H. Kobayashi K. (2017). Rapid and stable changes in maturation-related phenotypes of the adult hippocampal neurons by electroconvulsive treatment. *Mol Brain*. 10:8. 10.1186/s13041-017-0288-9 28253930 PMC5335812

[B14] Iori V. Frigerio F. Vezzani A. (2016). Modulation of neuronal excitability by immune mediators in epilepsy. *Curr. Opin. Pharmacol*. 26, 118–123. 10.1016/j.coph.2015.11.002 26629681 PMC4716878

[B15] Jessberger S. Aigner S. Clemenson G. D. Jr. Toni N. Lie D. C. Karalay O. et al. (2008). Cdk5 regulates accurate maturation of newborn granule cells in the adult hippocampus. *PLoS Biol.* 6:e272. 10.1371/journal.pbio.0060272 18998770 PMC2581629

[B16] Kataoka M. Yamamori S. Suzuki E. Watanabe S. Sato T. Miyaoka H. et al. (2011). A single amino acid mutation in SNAP-25 induces anxiety-related behavior in mouse. *PLoS One*. 6:e25158. 10.1371/journal.pone.0025158 21949876 PMC3176821

[B17] Kobayashi K. (2009). Targeting the hippocampal mossy fiber synapse for the treatment of psychiatric disorders. *Mol. Neurobiol*. 39, 24–36. 10.1007/s12035-008-8049-5 19130314

[B18] Kobayashi K. Suzuki H. (2007). Dopamine selectively potentiates hippocampal mossy fiber to CA3 synaptic transmission. *Neuropharmacology* 52, 552–561. 10.1016/j.neuropharm.2006.08.026 17049952

[B19] Kobayashi K. Ikeda Y. Suzuki H. (2011). Behavioral destabilization induced by the selective serotonin reuptake inhibitor fluoxetine. *Mol Brain*. 4:12. 10.1186/1756-6606-4-12 21410937 PMC3065414

[B20] Kobayashi K. Ikeda Y. Asada M. Inagaki H. Kawada T. Suzuki H. (2013). Corticosterone facilitates fluoxetine-induced neuronal plasticity in the hippocampus. *PLoS One*. 8:e63662. 10.1371/journal.pone.0063662 23675498 PMC3651130

[B21] Kobayashi K. Ikeda Y. Sakai A. Yamasaki N. Haneda E. Miyakawa T. et al. (2010). Reversal of hippocampal neuronal maturation by serotonergic antidepressants. *Proc. Natl. Acad. Sci. U S A*. 107, 8434–8439. 10.1073/pnas.0912690107 20404165 PMC2889553

[B22] Kobayashi K. Shikano K. Kuroiwa M. Horikawa M. Ito W. Nishi A. et al. (2022). Noradrenaline activation of hippocampal dopamine D1 receptors promotes antidepressant effects. *Proc. Natl. Acad. Sci. U S A*. 119:e2117903119. 10.1073/pnas.2117903119 35939697 PMC9388128

[B23] Krystal J. H. Karper L. P. Seibyl J. P. Freeman G. K. Delaney R. Bremner J. D. et al. (1994). Subanesthetic effects of the noncompetitive NMDA antagonist, ketamine, in humans. Psychotomimetic, perceptual, cognitive, and neuroendocrine responses. *Arch. Gen. Psychiatry*. 51, 199–214. 10.1001/archpsyc.1994.03950030035004 8122957

[B24] Kubová H. Mares P. Suchomelová L. Brozek G. Druga R. Pitkänen A. (2004). Status epilepticus in immature rats leads to behavioural and cognitive impairment and epileptogenesis. *Eur. J. Neurosci*. 19, 3255–3265. 10.1111/j.0953-816X.2004.03410.x 15217382

[B25] Lewis D. A. Levitt P. (2002). Schizophrenia as a disorder of neurodevelopment. *Annu. Rev. Neurosci*. 25, 409–432. 10.1146/annurev.neuro.25.112701.142754 12052915

[B26] Maglóczky Z. Halász P. Vajda J. Czirják S. Freund T. F. (1997). Loss of Calbindin-D28K immunoreactivity from dentate granule cells in human temporal lobe epilepsy. *Neuroscience* 76, 377–385. 10.1016/s0306-4522(96)00440-x 9015323

[B27] Marchal C. Mulle C. (2004). Postnatal maturation of mossy fibre excitatory transmission in mouse CA3 pyramidal cells: A potential role for kainate receptors. *J. Physiol*. 561, 27–37. 10.1113/jphysiol.2004.069922 15358807 PMC1665334

[B28] Martinian L. Catarino C. B. Thompson P. Sisodiya S. M. Thom M. (2012). Calbindin D28K expression in relation to granule cell dispersion, mossy fibre sprouting and memory impairment in hippocampal sclerosis: A surgical and post mortem series. *Epilepsy. Res*. 98, 14–24. 10.1016/j.eplepsyres.2011.08.011 21920703

[B29] Mori-Kawakami F. Kobayashi K. Takahashi T. (2003). Developmental decrease in synaptic facilitation at the mouse hippocampal mossy fibre synapse. *J. Physiol*. 553, 37–48. 10.1113/jphysiol.2003.045948 12963803 PMC2343498

[B30] Mulkey R. M. Endo S. Shenolikar S. Malenka R. C. (1994). Involvement of a calcineurin/inhibitor-1 phosphatase cascade in hippocampal long-term depression. *Nature* 369, 486–488. 10.1038/369486a0 7515479

[B31] Murano T. Hagihara H. Tajinda K. Takao K. Takamiya Y. Katoh K. et al. (2026). Repetitive neuronal activation regulates cellular maturation state via nuclear reprogramming. *Nat. Commun*. 17:5881. 10.1038/s41467-026-74202-w 42469214 PMC13379389

[B32] Nakazawa K. Sapkota K. (2020). The origin of NMDA receptor hypofunction in schizophrenia. *Pharmacol. Ther*. 205:107426. 10.1016/j.pharmthera.2019.107426 31629007 PMC6981256

[B33] Ohira K. Hagihara H. Toyama K. Takao K. Kanai M. Funakoshi H. et al. (2010). Expression of tryptophan 2,3-dioxygenase in mature granule cells of the adult mouse dentate gyrus. *Mol. Brain*. 3:26. 10.1186/1756-6606-3-26 20815922 PMC2945337

[B34] Ohira K. Kobayashi K. Toyama K. Nakamura H. K. Shoji H. Takao K. et al. (2013). Synaptosomal-associated protein 25 mutation induces immaturity of the dentate granule cells of adult mice. *Mol. Brain*. 6:12. 10.1186/1756-6606-6-12 23497716 PMC3605216

[B35] Ohshima T. Ogura H. Tomizawa K. Hayashi K. Suzuki H. Saito T. et al. (2005). Impairment of hippocampal long-term depression and defective spatial learning and memory in p35 mice. *J. Neurochem*. 94, 917–925. 10.1111/j.1471-4159.2005.03233.x 15992381

[B36] Overstreet-Wadiche L. S. Bromberg D. A. Bensen A. L. Westbrook G. L. (2006). Seizures accelerate functional integration of adult-generated granule cells. *J. Neurosci*. 26, 4095–4103. 10.1523/JNEUROSCI.5508-05.2006 16611826 PMC6673901

[B37] Pallas-Bazarra N. Kastanauskaite A. Avila J. DeFelipe J. Llorens-Martín M. (2017). GSK-3β Overexpression. *Front Neuroanat*. 11:18. 10.3389/fnana.2017.00018 28344548 PMC5344922

[B38] Peineau S. Nicolas C. S. Bortolotto Z. A. Bhat R. V. Ryves W. J. Harwood A. J. et al. (2009). A systematic investigation of the protein kinases involved in NMDA receptor-dependent LTD: Evidence for a role of GSK-3 but not other serine/threonine kinases. *Mol. Brain*. 2:22. 10.1186/1756-6606-2-22 19583853 PMC2715398

[B39] Radic T. Frieß L. Vijikumar A. Jungenitz T. Deller T. Schwarzacher S. W. (2017). Differential postnatal expression of neuronal maturation markers in the dentate gyrus of mice and rats. *Front. Neuroanat*. 11:104. 10.3389/fnana.2017.00104 29184486 PMC5694555

[B40] Rosenberg G. (2007). The mechanisms of action of valproate in neuropsychiatric disorders: Can we see the forest for the trees? *Cell Mol. Life Sci*. 64, 2090–2103. 10.1007/s00018-007-7079-x 17514356 PMC11149473

[B41] Sanches M. Keshavan M. S. Brambilla P. Soares J. C. (2008). Neurodevelopmental basis of bipolar disorder: A critical appraisal. *Prog. Neuropsychopharmacol Biol. Psychiatry*. 32, 1617–1627. 10.1016/j.pnpbp.2008.04.017 18538910

[B42] Scharfman H. E. (2019). The dentate gyrus and temporal lobe epilepsy: An “exciting”. *Era. Epilepsy Curr*. 19, 249–255. 10.1177/1535759719855952 31232111 PMC6891841

[B43] Schmidt-Hieber C. Jonas P. Bischofberger J. (2004). Enhanced synaptic plasticity in newly generated granule cells of the adult hippocampus. *Nature*. 429, 184–187. 10.1038/nature02553 15107864

[B44] Shin R. Kobayashi K. Hagihara H. Kogan J. H. Miyake S. Tajinda K. et al. (2013). The immature dentate gyrus represents a shared phenotype of mouse models of epilepsy and psychiatric disease. *Bipolar. Disord*. 15, 405–421. 10.1111/bdi.12064 23560889 PMC3752967

[B45] Sun D. Mei L. Xiong W. C. (2023). Dorsal dentate gyrus, a key regulator for mood and psychiatric disorders. *Biol. Psychiatry* 93, 1071–1080. 10.1016/j.biopsych.2023.01.005 36894487

[B46] Takao K. Kobayashi K. Hagihara H. Ohira K. Shoji H. Hattori S. et al. (2013). Deficiency of schnurri-2, an MHC enhancer binding protein, induces mild chronic inflammation in the brain and confers molecular, neuronal, and behavioral phenotypes related to schizophrenia. *Neuropsychopharmacology* 38, 1409–1425. 10.1038/npp.2013.38 23389689 PMC3682135

[B47] Tavitian A. Song W. Schipper H. M. (2019). Dentate gyrus immaturity in schizophrenia. *Neuroscientist* 25, 528–547. 10.1177/1073858418824072 30674225

[B48] Wigström H. Gustafsson B. (1983). Large long-lasting potentiation in the dentate gyrus in vitro during blockade of inhibition. *Brain Res*. 275, 153–158. 10.1016/0006-8993(83)90428-6 6313124

[B49] Yamasaki N. Maekawa M. Kobayashi K. Kajii Y. Maeda J. Soma M. et al. (2008). Alpha-CaMKII deficiency causes immature dentate gyrus, a novel candidate endophenotype of psychiatric disorders. *Mol. Brain*. 1:6. 10.1186/1756-6606-1-6 18803808 PMC2562999

[B50] Zeiss C. J. (2021). Comparative milestones in rodent and human postnatal central nervous system development. *Toxicol. Pathol.* 49, 1368–1373. 10.1177/01926233211046933 34569375

[B51] Zucker R. S. Regehr W. G. (2002). Short-term synaptic plasticity. *Annu. Rev. Physiol*. 64, 355–405. 10.1146/annurev.physiol.64.092501.114547 11826273

