# Supplementary material for: Developmental changes in activity-dependent plasticity of maturation-related neuronal phenotypes
Source: Front Synaptic Neurosci. 2026 Oct 15;18:1926357. doi: 10.3389/fnsyn.2026.1926357 (PMC13716739; doi:10.3389/fnsyn.2026.1926357)
Supplement: Supplementary file 1 [file Image_1.pdf]

Supplementary Material

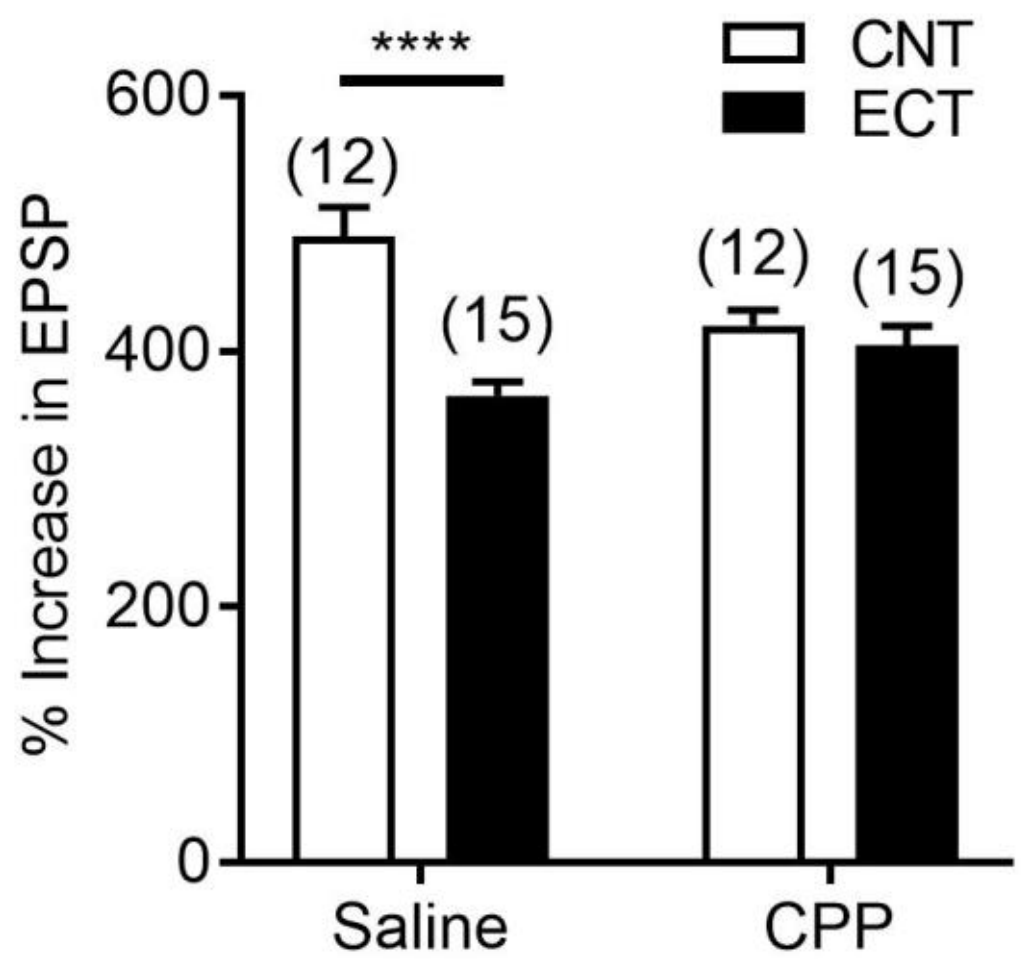

Supplementary Figure 1. NMDA receptor-dependent induction of dematuration in adult mice. The NMDA receptor antagonist CPP (40 mg/kg) or saline was intraperitoneally injected 30 min before each ECT. Frequency facilitation at the MF synapses was reduced by ECT in the saline-injected mice, but not in CPP-injected mice (two-way ANOVA [ECT effect: $F_{1,50} = 19.74$, $p < 0.0001$; CPP effect: $F_{1,50} = 0.9024$, $p = 0.3467$; interaction: $F_{1,50} = 11.82$, $p = 0.0012$] followed by Sidak's test [****$p < 0.0001$]).
